# Esmeralda Peach (*Prunus persica*) Fruit Yield and Quality Response to Nitrogen Fertilization

**DOI:** 10.3390/plants11030352

**Published:** 2022-01-27

**Authors:** Gilberto Nava, Carlos Reisser Júnior, Léon-Étienne Parent, Gustavo Brunetto, Jean Michel Moura-Bueno, Renan Navroski, Jorge Atílio Benati, Caroline Farias Barreto

**Affiliations:** 1 Empresa Brasileira de Pesquisa Agropecuária—Embrapa Clima Temperado, Pelotas 96010971, Brazil; gilberto.nava@embrapa.br (G.N.); carlos.reisser@embrapa.br (C.R.J.); 2 Soil Science Department, Federal University of Santa Maria (UFSM), Santa Maria 97105900, Brazil; Leon-Etienne.Parent@fsaa.ulaval.ca (L.-É.P.); brunetto.gustavo@gmail.com (G.B.); 3 Department of Soils and Agrifood Engineering, Laval University, Quebec City, QC G1V 0A6, Canada; 4 Fruit Tree Department, Federal University of Pelotas (UFPEL), Pelotas 96010610, Brazil; navroski@outlook.com (R.N.); jorgeatiliobenati@hotmail.com (J.A.B.); carol_fariasb@hotmail.com (C.F.B.)

**Keywords:** fruit acidity, antioxidants, climatic features, nitrogen dosage, nutrient balances, fruit, firmness, total soluble solids

## Abstract

‘Esmeralda’ is an orange fleshed peach cultivar primarily used for juice extraction and secondarily used for the fresh fruit market. Fruit yield and quality depend on several local environmental and managerial factors, mainly on nitrogen, which must be balanced with other nutrients. Similar to other perennial crops, peach trees show carryover effects of carbohydrates and nutrients and of nutrients stored in their tissues. The aims of the present study are (i) to identify the major sources of seasonal variability in fruit yield and qu Fruit Tree Department of Federal University of Pelotas (UFPEL), Pelotas 96010610ality; and (ii) to establish the N dose and the internal nutrient balance to reach high fruit yield and quality. The experiment was conducted from 2014 to 2017 in Southern Brazil and it followed five N treatments (0, 40, 80, 120 and 160 kg N ha^−1^ year^−1^). Foliar compositions were centered log-ratio (clr) transformed in order to account for multiple nutrient interactions and allow computing distances between compositions. Based on the feature ranking, chilling hours, degree-days and rainfall were the most influential features. Machine learning models k-nearest neighbors (KNN) and stochastic gradient decent (SGD) performed well on yield and quality indices, and reached accuracy from 0.75 to 1.00. In 2014, fruit production did not respond to added N, and it indicated the carryover effects of previously stored carbohydrates and nutrients. The plant had a quadratic response (*p* < 0.05) to N addition in 2015 and 2016, which reached maximum yield of 80 kg N ha^−1^. In 2017, harvest was a failure due to the chilling hours (198 h) and the relatively small number of fruits per tree. Fruit yield and antioxidant content increased abruptly when foliar clr_Cu_ was >−5.410. The higher foliar P linearly decreased total titratable acidity and increased pulp firmness when clr_P_ > 0.556. Foliar N concentration range was narrow at high fruit yield and quality. The present results have emphasized the need of accounting for carryover effects, nutrient interactions and local factors in order to predict peach yield and nutrient dosage.

## 1. Introduction

Fruit appearance, firmness, price, epidermis color and fruit size are consumers’ main criteria for peaches purchase [1]; consumers would rather buy fruits with yellow flesh and red epidermis. Fruit general appearance and aroma are appealing at first, but perceptions about previously consumed fruit flavor and texture have impact on consumers’ choices [2]. Total soluble solids (TSS) reflect fruit sweetness, whereas total titratable acidity (TTA) accounts for juice acidity and sourness. Assumingly, antioxidants that include phenolic compounds and carotenoids support human health [3].

Regional climatic conditions influence the geographical distribution of peach production [4]. Rootstock and cultivar must conform with regional biotic and abiotic conditions [5,6,7,8]. Low-chilling peach cultivars commonly used in subtropical environments require less than 150 chilling units, whereas this number reaches 350–650 chilling units for medium chilling cultivars [4,9]. Irrigation and pruning methods have impact on peach yield and fruit quality, as well as on disease incidence [10]. Tillage, fertilization and pruning removal influence the C cycle in peach orchards [11].

Fertilization, mainly with nitrogen (N), has impact on the composition of foliar nutrients, as well as on peach yield and quality [12,13,14,15,16,17]. Nitrogen fertilization extends the fruit development period, and it explains these fruits’ capacity to sink carbohydrates and nutrients [18]. Nitrogen (N) shortage decreases flower buds’ quality, and it affects the effective fruiting supported by them [19], such a shortage also impairs peach root system growth, affects water and nutrients uptake, and decreases root capacity to sustain shoot growth [20,21] and fruit production [22,23,24]. On the other hand, excessive N doses and high N levels in plant tissue result in vegetative overgrowth, increase in labor costs with green and winter pruning, decrease in fruit quality and increase in disease incidence [23,25,26]. Excessive N applications also lead to N loss due to leaching or runoff, which pose a risk for neighboring water contamination [27,28,29]. Site-specific conditions must be successfully combined to enable high crop yield and quality, as well as sustainable environment.

Based on the close examination of N fertilization trials carried out in Southern Brazil, optimum N doses depend on climatic conditions, soil type, soil organic matter content, soil management, pruning, fruit thinning, cultivar and plantation density [12,13,26,30,31]. Peach orchards are often planted in sandy soils, with low in organic matter (OM) content, which likely supply insufficient amounts of nitrogen to plants [28,32,33]. Therefore, nitrogen fertilization is required to maintain internal N reserves and crop yield [34,35]. Nitrogen (N) recommendations mostly rely on tissue tests [36,37] and on concentration sufficiency ranges [22,35]. The log-ratio transformation of nutrient concentrations is a mathematically suitable multi-ratio expression used to compute Euclidean multivariate distances and to run machine learning models [38,39]. 

Similar to other perennials [40], peach trees show carryover effects of stored carbohydrates and nutrients that have impact on fruit yield and quality, as well as on nutrient dosage. Carbohydrate storage can be documented by both pruning and previous yields. Nutrient storage can be reported based on foliar nutrients from the previous year. Key features can be processed as unique combinations based on using machine learning algorithms to derive nutrient standards and predict site-specific tree performance and optimal nutrient dosage.

We hypothesized that previous environmental and managerial factors, as well as nutrient and carbohydrate storage, have impact on both peach tree yield and foliar tissue composition. The aims of the present study were (i) to identify the major sources of seasonal variability in fruit yield and quality; (ii) to establish the N dose and the internal nutrient balance required for peach trees to reach high fruit yield and quality; and (iii) to derive foliar N standards at high yield and quality levels.

## 2. Materials and Methods

### 2.1. Study Site

**Figure 1:**
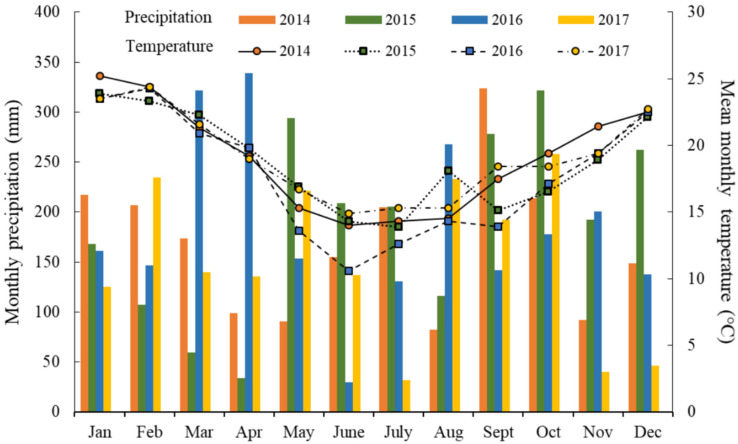
Mean monthly rainfall (mm) and air temperature (°C) at the experimental site.

The study was carried out in a commercial ‘Esmeralda’ orchard in Morro Redondo County (31°31′55′′ S, 52°35′37′′ W—altitude 200 m), Rio Grande do Sul State, Southern Brazil. The soil in the site was classified as Ultisol, based on the US Soil Taxonomy [41]. Soil contained 180 g clay dm^−3^. Climate in the region is of the “Cfa” type, according to Köppen’s classification [42], with humid temperate climate and hot summers. The region has annual temperature of 18 °C, precipitation of 1509 mm and relative humidity of 78.8%, on average. Monthly precipitation and mean temperature data recorded during the experiment are presented in Figure 1 [43].

‘Esmeralda’ is a low-chill cultivar (nearly 350 chilling hours are optimally required) with clingstone and orange non-melting flesh [44]. Capdeboscq was the used rootstock. N, P and K fertilization followed the regional guidelines during the first five years after trees were planted [45]. Plants only received N at doses ranging from 50 to 80 kg N ha^−1^ for the first three years. They received 90 kg N ha^−1^, 20 kg P ha^−1^ and 50 kg K ha^−1^ in the fourth and fifth years. The N trial started in 2014; treatments consisted of 0, 40, 80, 120 and 160 kg N ha^−1^ year^−1^ applied at urea form (44% N) on soil surface, without incorporation, 2 m away from the planting line. Half of the N was applied at the beginning of the flowering period and the other half at fruit thinning. Plots also received 60 kg K ha^−1^ and 20 kg P ha^−1^ in single application at the beginning of sprouting period (second half of July).

The study followed a randomized block design with four repetitions. Experimental units comprised four plants, with 6-m spacing between rows, and 1.5 m spacing between plants. There were 1111 plants per ha^−1^. The “Y system”, which does not allow plants to grow more than 2.5 m in height, was the training system of choice [46]. The orchard was not irrigated.

Harvest was carried out on two central plants, at three different occasions (harvests 1, 2 and 3). Fruit maturity was reached at pulp firmness of 1.7–3.6 Newton and total soluble solids (TSS) of 9.0–14.0 °Brix. Fruits were counted and weighed. Total number of fruits per tree, mean fruit mass and fruit yield were recorded.

### 2.2. Soil Analysis

Soil samples (12 subsamples of the composite sample) were collected with the aid of a hand steel auger, in the 0–0.20 m soil layer, across the experimental area in 2009, and in each plot in 2017. Soil samples were air-dried and ground until reaching particles smaller than 2 mm, before analyses [47]: pH in 1:1 soil-to-water volumetric ratio; clay by densimetry; Mehlich-1 ex-traction for P, K, Cu, Zn and Fe; KCl extraction for Mn; and hot water extraction for B. Elements were quantified through plasma-emission spectroscopy (ICP-OES—Optima^®^ 8300, Perkin Elmer, Waltham, MA, USA). Total carbon was quantified through dichromate oxidation (Walkley–Black) and multiplied by 1.724 in order to assess organic matter content [48].

Soil acidity was corrected with dolomitic limestone in order to raise pH to 6.0 three months before seedlings’ planting in April 2009. Phosphorus (P) and K levels in the soil were corrected to reach high level of both nutrients, according to [45]. Limestone, triple superphosphate and potassium chloride were broadcast applied and incorporated into the 0–0.30 m soil layer after a whole sequence of subsoiling, plowing and harrowing. Soil analyses at experiment onset and at the end of it are shown in Table 1.

### 2.3. Leaf Collection and Analysis, and Fruit Yield

In total, 40 leaf samples per experimental unit were collected from the middle part of the branches, at tree mid-height, around the plant and composited in November 2014, 2015, 2016 and 2017 (approximately 100 days after full bloom). Leaves were gently cleaned in distilled water, oven-dried at 65 °C and ground to particles smaller than 1 mm. Nitrogen (N) content was determined by combustion by using the CHN-S analyzer (TruSpec CHN-S LECO, St. Joseph, MI, USA). Tissue samples were digested in a mix of nitric (3 mL—concentration 65%) and perchloric (1 mL—concentration 70%) acids and analyzed through colorimetry for P and B, through flame photometry for K and through atomic absorption spectrophotometry for Ca, Mg, Cu, Fe, Mn and Zn [47].

### 2.4. Fruit Parameters

A sample with 30 fruits from the two central plants of each repetition was collected in each plot for fruits’ physicochemical evaluations. The following parameters were evaluated in fruits in this sample: fruit firmness was assessed in N by using texture analyzer (TA.XT plus^®^—Stable Micro Technologies Texture Systems, Godalming, UK) with 2-mm tip, at 5 g force and speed of 5 mm s^−1^ [49]. Hue and chroma were measured through reflectometry (Minolta CR-400, Ramsey, NJ, USA). Color became more intense as chromaticity increased and duller as it decreased [2]—hue represents tint of color. Thereafter, fruits were crushed to determine TSS contents in the juice (°Brix) by using a portable digital refractometer (PAL-1, Atago, Bellevue, WA, USA) with temperature control. TTA (mg of citric acid per 100 mL) was quantified by titration with 0.1 N NaOH and by using phenolphthalein as indicator [49]. Pulp pH was potentiometrically measured. After peeling, fresh fruits were analyzed for phenolic compounds (mg chlorogenic acid per 100 g), according to [50]; standardized against malic acid [51] and carotenoids (mg per 100 g), according to [52]; and antioxidant activity (mg trolox-equivalent per 100 g fruit), according to [53].

### 2.5. Meteorological Data

Climatic conditions, at local scale, had impact on fruit transpiration, growth and phloem transport [54]. Meteorological data were collected at the closest weather station (Embrapa, Pelotas, Brazil). Rainfall, number of chilling hours (>7.2 °C), total rainfall and cumulated growing degree-days between bloom and harvest were the climatic indices of choice [55]. Full bloom was observed on 29 July 2014; on 1 August 2015; on 3 August 2016 and on 18 July 2017. Fruits were harvested between 30 November and 16 December 2014; between 2 and 20 December 2015; between 5 and 22 December 2016; and between 16 and 30 November 2017.

The number of cumulated growing degree days (GDD) was computed by following two calculation methods [56]. Based on Method 1, a single minimum base temperature ($T_{base}$) is provided to support plant growth (Equation (1)):

(1) $$GDD_{1}=\sum_{i=1}^{t}\left(T_{min}+T_{max}\right)/2-T_{base}$$

where in, *i =* 1→*t* represents the production period duration, $\left(T_{min}+T_{max}\right)/2-T_{base}$ is daily mean temperature between minimum ($T_{min}$) and maximum ($T_{max}$) temperatures. Mean daily temperature is adjusted to base temperature in Method 1.

Both lower threshold temperature (LT) and upper threshold temperature (UT) values are provided in Method 2, as well as daily minimum and maximum temperatures are conditionally adjusted, based on Equation (2):

(2) $$GDD_{2}=\sum_{i=1}^{t}\left(T_{min}+T_{max}\right)/2-T_{base}$$

submitted to the following conditions for LT and UT:

If $T_{max}<T_{LT};T_{max}=T_{LT}$, 

If $T_{min}<T_{LT};T_{min}=T_{LT}$,

If $T_{max}>T_{UT};T_{max}=T_{UT}$, and

If $T_{min}>T_{UT};T_{min}=T_{UT}$

Minimum and maximum temperatures were adjusted, in separate, to lower threshold temperature ($T_{LT}$) or upper threshold temperature ($T_{UT}$) before computing $GDD_{2}$. Reference [31] used 7 °C as lower threshold temperature to trigger peach tree growth—which is also called “base temperature” ($T_{base}$)—and 35 °C as upper threshold temperature—peach tree growth is assumed to cease at temperatures higher than 35 °C. In this case, the minimum number of degree-days is zero, wherein $T_{min}\leq 7\;{}^{\circ}C;$ and maximum number of degree-days is 28, wherein $T_{max}\geq 35\;{}^{\circ}C$.

$GDD_{1}$ was highly correlated to $GDD_{2}$ (r = 0.99). $GDD_{2}$ was selected based on [31], it was computed between full bloom and harvest in order to indicate the time elapsed to reach fruit maturity.

### 2.6. Statistical Analysis

Data were statistically analyzed in the SAS Statistical package, version 6.08 [57]. Modeling was supplemented by using the “rjags” package [58], in R statistical environment [59]. Hierarchical Bayesian analysis adjusted the regression models. Monte Carlo simulation was performed by using Markov chains (MCMC) and the Gibbs sampling algorithm [60]. Frequency density analysis was performed assuming 90% confidence intervals to determine borderline concentrations (FS) and the highest density of nutrients occurrence (NC).

Machine learning (ML) models were run in the Orange data mining free software, vs. 3.29.3 (https://orange.biolab.si/download/#windows (accessed on 1 July 2021)). Nutrients were centered logarithmically prior to the models being run [39,40]. Centered log ratios were computed as follows: $clr_{x_{i}}=ln\left(x_{i}/G\right)$, wherein $x_{i}$ is the ith component of the D-parts foliar composition (N, P, K, Ca, Mg, B, Cu, Zn, Mn, Fe, F_v_). Filling value F_v_ was computed based on the difference between the measurement unit (1000 g) and the sum of quantified nutrients expressed in g per 1000 g. Geometric mean G was computed across components. Models were trained by using stratified cross-validation (k = 10).

The classification mode about the cutoff value for target variables allowed separating high from low crop performance categories in order to set true negative specimens apart and to compute foliar nutrient standards. Data were partitioned in the confusion matrix as true negative (high predicted and high actual targets), true positive (low predicted and low actual targets), false negative (high predicted and low actual targets) and false positive specimens (low predicted and high actual targets).

The machine learning model ML1, which was chosen for the current year, used the 80-year observations in the data set. Model ML1 was elaborated to compute statistics for true negative specimens (high-yielding and nutritionally balanced specimens) across time periods (t) 2014–2017, according to Equation (3):

(3) $$Target_{t}=f\left(Features_{t}\right)$$

Foliar nutrient standards were computed as centered log ratios of true negative specimens. The corresponding nutrient concentration ranges were computed by back-transforming clr confidence interval values (*p* = 0.01 for two-tail *t* test) as follows:

(4) $$C_{x_{i}}=exp\left(clr_{x_{i}}\right),i=1\;to\;D$$

(5) $$S^{D}=\left\{\frac{C_{x_{1}}}{Sum}\times \kappa ,\frac{C_{x_{2}}}{Sum}\times \kappa ,\ldots ,\frac{C_{x_{D}}}{Sum}\times \kappa \right\}$$

where in, $C_{x_{i}}$ is the primary back-transformation, $S^{D}$ is the compositional simplex closed to measurement unit after adjusting κ to measurement unit, such as 1000 g kg^−1^ or 10^6^ mg kg^−1^.

Machine learning model ML2, which was chosen to make predictions, included previous-year foliar nutrient compositions and yield in order to account for nutrients and carbohydrate storage, respectively. Model ML2 was elaborated as follows, by using time t+1 to make predictions, according to Equation (6):

(6) $$Target_{t+1}=f\left(Features_{t}\right)+Target_{t}+Fertilization_{t}$$

Model ML2 was trained by using 60 observations (2014–2016); it was tested with 20 observations, in 2017, in order to compare predicted to actual yield response curves.

## 3. Results

### 3.1. Fruit Yield and Yield Components

**Figure 2:**
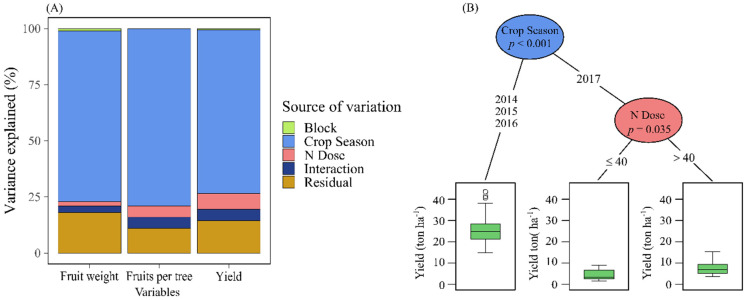
Variance proportion explained by driving variables (**A**). The conditional inference tree shows the effect of cropping season and N dosage on fruit yield (**B**).

There were large variations in climatic conditions between years (Figure 1). Cropping season contribution to total variance was 73% for fruit yield, 79% for number of fruits per plant and 76% for mean fruit weight (Figure 2A). Nitrogen (N) dose contributed to 7% total variation in fruit yield (Figure 2B).

The 2014, 2015 and 2016 seasons shared similar yield. Year 2017 was a commercial failure. One of the factors that stood out the most in 2017 in relation to other years was the number of chilling hours, being the smallest in 2017 compared to other years (219 CH in 2015, 348 CH in 2016, and 367 CH in 2014). Nitrogen (N) fertilization sustained fruit yield from 2014 to 2016 (Table 2). Fruit yield was not influenced by N treatments in 2017. The highest yields were recorded at 80 kg N ha^−1^, in 2014, 2015 and 2016. Yield recorded non-linear response to N addition in 2015 and 2016, due to the large number of fruits per tree.

### 3.2. Fruit Yield and Quality Impacted by Key Features in Machine Learning Models

Meteorological indices, foliar mineral compositions and previous production season yield were the features of choice to run machine learning models. There were 11 foliar components, including the filling value; thus, 23 features accounted for nutrients (tissue analysis) and carbohydrates (yield) cumulated in the previous season. Meteorological features were reduced to a minimum set due to the limited total number of observations (80). $GDD_{2}$, total rainfall and number of chilling hours were retained as meteorological features.

Features were scored by ranking their association with the target variables by using the univariate regression ranking method (Table 3). Dominant features for most target variables were the meteorological features of choice. All nutrients had impact on target variables, to some degree.

Features were scored by ranking their association with target variables by using the univariate regression ranking method (Table 3). All nutrients had impact on target variables, to some extent. Meteorological features were dominant for most target variables. Chilling hours had impact on yield, pulp firmness, fruit hue and carotenoid content. Yield was low at 198 chilling hours and high at 219 chilling hours. More than 1400 GDD2 increased pulp firmness. Rainfall higher than 700 mm increased hue and decreased chroma at rates higher than 850 mm.

KNN and SGD were the best performing models to predict fruit yield and quality indices (Table 4). Epidermis and flesh firmness, fruit hue, chroma, TSS, TTA and total antioxidant activity were modelled as target variables. True negative specimens were outnumbered (10–17) for carotenoids and phenolic compounds in order to derive foliar nutrient standards at high target levels. The number of true negative specimens (32–59) sufficed for most target variables to compute nutrient standards.

**Figure 3:**
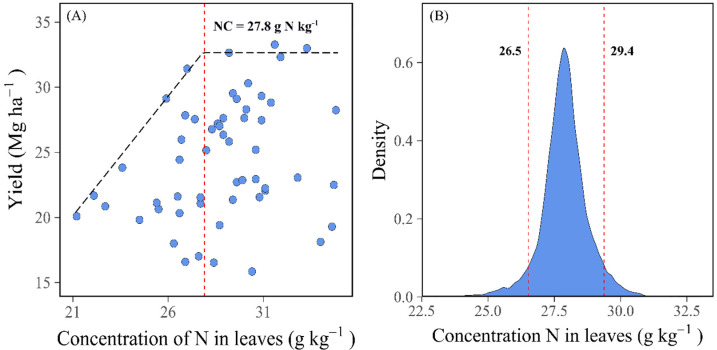
Association between relative yield and foliar N concentration (**A**). Histogram of foliar N concentration density frequency to indicate sufficiency range (**B**).

Nutrient ranges presented as mean and standard deviation of clr values recorded for the associated true negative specimens are shown in Table 5. Obviously, high fruit quality can be attained within a narrow range of foliar N concentrations and within much wider concentration ranges for other nutrients. The critical N concentration value was close to 27.8 g N kg^−1^ when the MCMC-Gibbs simulation was used (Figure 3A), as well as to the sufficiency range (SR) of N between 26.5 to 29.4 g N kg^−1^, based on the density distribution function (Figure 3B).

**Figure 4:**
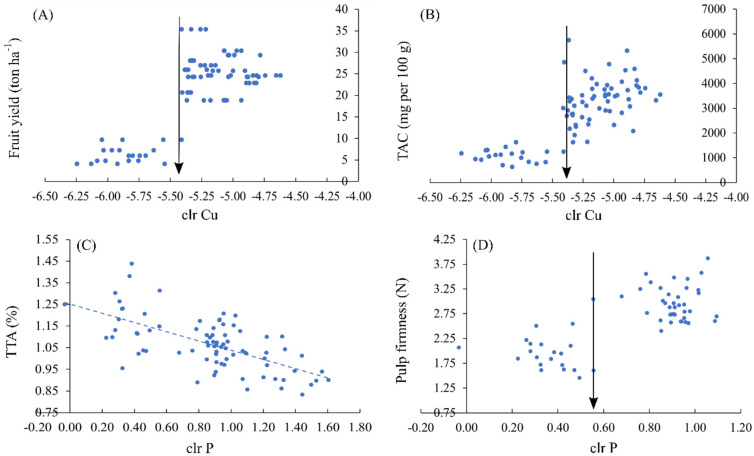
Association between foliar Cu and P balances (clr values), and fruit indices (**A**–**D**). Critical values are indicated by arrows. Trend is indicated by dotted line.

Cu and P were the most influential nutrients to fruit yield and quality. Critical foliar clr_Cu_ value was −5.410 for both yield and antioxidant content (Figure 4A,B). Foliar P was closely related to pulp firmness and TTA. Higher foliar P linearly decreased TTA (Figure 4C). Pulp firmness increased when the clr_P_ value exceeded 0.556 (Figure 4D).

### 3.3. Predicted vs. Actual Yield in 2017

**Figure 5:**
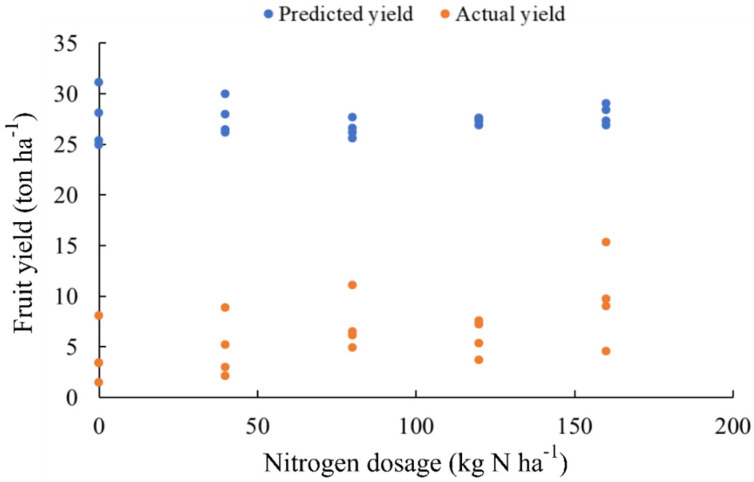
Predicted and actual fruit yield recorded in 2017 based on foliar nutrient composition and previous yield—as priors in regression Model ML2. Gradient Boosting was used as learner.

Crop response to N addition was significantly quadratic in 2015 and 2016, but it was small in 2017 in comparison to model prediction (Figure 5). Model ML2 predicted 100% probability of having high yields. The low yield recorded in 2017 was attributed to adverse climatic conditions. There was no need of N fertilization in 2017, despite the high yield potential predicted by ML2. Applying state recommendation (80 kg N ha^−1^) for soils presenting low organic matter content, without taking into consideration the carryover effects of previous yield and nitrogen storage, would lead to economic and environmental losses.

## 4. Discussion

### 4.1. Climatic Features

Climatic features strongly affected peach fruit yield and quality, as shown in other studies [61,62]. The interest for low-chill cultivars in Brazil is exacerbated by climate change, which is bringing warmer winters and has impact on dormancy and on leafing and blooming uniformity [63]. Brazilian low-chill oftentimes makes cultivars require 200–300 chilling hours to emerge from dormancy [64]. ‘Esmeralda’ apparently required more than 198 chilling hours, if number of chilling hours was the limiting factor, whereas 219 chilling hours were likely sufficient to reach high fruit yield and quality levels. The small number of chilling hours apparently led to low commercial yield in 2017, in the present study.

### 4.2. Foliar Nutrients

Peach cultivars surveyed in Southern Brazil showed differential nutritional profiles, and it may lead to cultivar-specific fertilization [65]. ‘Esmeralda’ nutrient ranges (Table 4) were narrow and differed from nutrient standards suggested for Southern Brazil [37], or elaborated among peach cultivars surveyed in Rio Grande do Sul State, Brazil [39]. Leaf and stem compositions are influenced by several environmental, managerial and physiological factors [39,66] that change from study to study. This finding implies nutrient diagnosing based on regional standards is at risk of application in a specific peach orchard. On the other hand, local factors can be processed by machine learning models to solve site-specific problems based on well-documented data sets [67].

The apparent imbalance in foliar P (clr_P_) to reach high pulp firmness in 2017 cannot be attributed to soil P, which remained at very high soil test level throughout the experiment (Table 1). Similarly, median foliar P concentration was 4.6 g P kg^−1^ in 2014, 2.8 g P kg^−1^ in 2015, 1.8 g P kg^−1^ in 2016 and 3.0 g P kg^−1^ in 2017, which would suffice to achieve high crop yield and quality (Table 4). Firmer pulp apparently required more P in comparison to fruit yield and other fruit quality indices; however, higher foliar P accounted for more sour fruits. Higher pulp firmness and more sourness have been associated with fruit immaturity [68,69].

Foliar Cu had likely impact on fruit yield and TAC, to a large extent (Figure 3). Cu is commonly associated with both plant yield and antioxidant activity [70,71]. Median foliar Cu concentration was 10 mg Cu kg^−1^ in 2014, 7 mg Cu kg^−1^ in 2015, 6 mg Cu kg^−1^ in 2016 and 3 mg Cu kg^−1^ in 2017. Cu concentration in 2017 was apparently too low to reach high crop yield and TAC (Table 4).

Foliar N concentration ranges recorded for ‘Esmeralda’ were lower than the 33 to 45 g N kg^−1^ suggested for the region [37]. State standards may lead to N over-fertilization and nitrate leaching in ‘Esmeralda’ orchards, and it could have potential impact on water quality [28,29]. Excessive vegetative growth has impact on fruit yield and quality [23,25], as well as the incidence of fungal diseases due to decreased air circulation within the canopy [72]. When N addition exceeds its demand by peach trees, vegetative growth is stimulated and it limits sunlight dispersion throughout the canopy [23,35]. In addition, longer hours must be devoted to both pruning and fungicide applications [26,35].

### 4.3. Fruit Yield

Nitrogen (N) stored in plant tissues may not be enough to sustain high crop yield, adequate foliar N concentration, fruit yield and vegetative growth [66]. Effective fruiting is promoted when the ovule remains active during the necessary period-of-time to support proper tree nutrition [73]. Fruit yield reached 27 ton ha^−1^ at zero-N application in 2014; this number was much higher than the mean fruit yield of 10.1 tons ha^−1^ recorded for Rio Grande do Sul [74]. Nitrogen (N) addition of 80 kg N ha^−1^ year^−1^ did not have impact on crop yield in 2014, but it did it in 2015 and 2016. However, two consecutive seasons under the 80 kg N ha^−1^ regime built high N reserves in plant tissue and it led to no apparent response to N addition in 2017. State recommendation for soils containing less than 2.6% organic matter is 80 kg N ha^−1^ year^−1^ [37], but it led to no response to N addition in 2014 and 2017—this finding indicates carryover effects. This outcome supports the need of feature-specific N recommendation, given information on previous yield and foliar nutrients. The 2017 prediction assumed 25–30 tons ha^−1^, but harvest was a commercial failure (Figure 5) in this year due to adverse climatic conditions. Nonetheless, the machine learning model provided fair estimates of potential damage due to harsh climatic conditions.

The large number of fruits per plant in 2016 (Table 2) could trigger alternate bearing by contributing to the reduced fruit yield recorded in 2017. Higher N dosage increased the number of fruits per tree, but fruit mass was not affected by it in most assessed harvests. Similarly, N applications to apple trees showed little effect on fruit size, but it influenced the number of fruits per tree [75]. With respect to stone fruits, a large number of fruits would have impact on the source-drain balance of nutrients and carbohydrates, a fact that led to competition between vegetative and reproductive plant parts, and to yield drop in the subsequent season [76,77]. Fruiting and fruit development decreased as flowers production also decreased [23,27]. Excessive fruit load in one crop can reduce vegetative growth and fruit set in the following cropping season [76]. Nevertheless, fruit yield predicted by Model ML2 did not support alternate bearing as the main cause of crop failure in 2017. The number of chilling hours (CH) in 2017 (198 CH) was relatively small for ‘Esmeralda’, which could require up to 350 CH, as observed in 2016, which was the most productive year.

Model ML2 predicted no need of N fertilization at high-yield level in 2017, and this finding indicates carryover effects and sufficient nutrient and carbohydrate storage. Fruit trees are very sensitive to shortage of carbohydrates and other resources [54], but they can store nutrients in their tissues over the years and show little response to added nutrients in the following season [77,78,79,80,81,82,83,84]. Nitrogen (N) reserves can be stored in roots, stems and branches (older than 1 year) [19,23]. Stored N can be redistributed by phloem flow [27], and this phenomenon is commonly observed in temperate fruit trees [66,79]. Soil organic matter and its prior fertilization can also supply nitrogen through the mineralization of organic N, roots and leftover residues [27,82,83].

### 4.4. Fruit Quality

Most studies on peach fruit quality have focused on individual quality features such as texture, aroma, color, size, sugar concentration and acidity [2]. Fruit appearance is the first factor assessed by field workers to start the harvesting period in commercial peach production systems. Skin redness (foreground color), background color and firmness are other benchmarks to assess optimal fruit maturity at harvest. Color is the feature most commonly associated with fruit maturity [84]. Taste is driven by the combination of sweetness and sourness, which is produced by sugars and organic acids. TSS indicates sweetness and TTA is a sourness indicator. Previous studies have linked consumer’s sweetness perception to TSS:TTA ratio [1]; however, this ratio is biased in favor of low-acid cultivars. Nevertheless, a collection of observable quality descriptors for individual cultivars is yet to be established. An ensemble of traits may include shape, skin and flesh color, texture type, volatile profile, and sugar and acid concentration [2,84]. The year-to-year variability in sugar concentration has been attributed to environmental effects, to the inherent variation among cultivars, and to their interaction [85].

Quality standards for yellow fleshed peaches in California have been set at 10% TSS, minimum [85]. In Italy, 10% is the minimum TSS suggested for the early season, 11% for the midseason and 12% for the late-season cultivars [86,87]. In France, quality indices of 10% TSS and 0.9% TTA were also suggested for low-acidity peaches [88]. Ripe peaches are considered ‘‘ready-to-eat’’ at 0.9–1.4 kgf flesh firmness [89]. Our cutoff target variables followed these guidelines.

Climate and crop load, along with individual cultivar differences, are the major components influencing fruit quality variability [90]. Climatic indices and several nutrients had impact on fruit quality at the study site. Machine learning methods can easily handle myriads of such factor combinations, which have impact on crop yield and quality, and on the selection of successful combinations. Controllable factors, such as nutrients, can be locally adjusted to reach high, high-quality fruit yield [69].

### 4.5. Building a Brazilian Data Set for Nutrient Management in Peach Orchards

Many other factors could sustain fruit yield and quality. Trunk diameter (20 cm) above soil surface, twig annual growth length, fruit diameter, fruit mass, luminescence, production per plant, number of fruits per plant, fruit mineral composition, summer or winter pruning biomass, canopy volume, plant height, number of buds per cm of shoot, leaf chlorophyll index, total leaf area and rust incidence [13,24,26] could be further documented to improve the accuracy of ML models. The larger the number of features, the larger must be the number of observations to be acquired [13,24,26,35,39].

Fully traceable growers, and reliably and ethically collected observational data, and other features, could be acquired to increase peach data set size and to improve fruit yield and quality predictions among cultivars, in addition to experimental data such as the herein used ones. The data set should be built uniformly for common use by stakeholders in order to continuously improve local fertilizer recommendations based on trustfully documented local conditions and successful cases.

## 5. Conclusions

Nitrogen fertilization increased the fruit yield of cultivar ‘Esmeralda’ by up to 54%. The number of fruits per plant was the parameter mostly influenced by N fertilization. Maximum fruit yield was obtained by applying 80 kg N ha^−1^ in 2015 and 2016, as suggested by state guidelines for soils presenting low organic matter content. No N fertilization was needed in 2014 and 2017, and this finding indicates that N fertilization should be adjusted to plant carbohydrates and nitrogen storage.

The foliar N range was narrow at high fruit yield and quality. The critical foliar N concentration to reach high yield was 28 g kg^−1^, and this value is low in comparison to the sufficiency range currently used in Southern Brazil. Nutrients could be balanced by using centered log ratios (clr) as nutrient indices to derive nutrient standards and critical values. Fruit yield and antioxidant content increased abruptly when foliar Cu clr_Cu_ value was >−5.410. Higher foliar P linearly decreased TTA and pulp firmness increased when foliar P clr_P_ value was >0.556.

State recommendation (80 kg N ha^−1^ year^−1^) appeared to be too high under the site-specific conditions in the current experiment. This finding supports feature-specific fertilizer recommendations set for crop yield and fruit quality, given information on previous carbohydrate reserves (previous yield) and site-specific foliar nutrient combinations (foliar analysis). The present research emphasized the need of accounting for carryover effects, nutrient interactions and local factors by acquiring and modelling massive experimental and observational data.

## Figures and Tables

**Table 1:** Soil properties at the beginning (3 weeks after lime and fertilizer incorporation in 2009) and at the end of the experiment in 2017. Properties in 2017 are median values across plots.

Year	pH	OM	Ca	Mg	P	K	Cu	Fe	Mn	Zn	B
		g kg^−1^	cmol_c_ kg^−1^		mg kg^−1^						
2009	5.8	21	3.0	0.95	23	64	0.53	2.8	6.5	2.8	0.20
2017	6.0	23	4.6	1.2	24	129	0.61	3.4	7.8	2.6	0.30

pH = pH in water (relation 1:1 soil/solution); OM = Organic matter.

**Table 2:** Mean N dosage effect on marketable fruit yield, number of fruits per plant and mean fruit mass in peach trees.

N Rate	2014	2015	2016	2017
kg N ha^−1^	Fruit yield (ton ha^−1^)			
0	27.0	18.9	20.7	4.1
40	24.3	18.9	26.0	4.8
80	29.4	30.4	35.4	7.3
120	24.6	24.6	28.1	6.0
160	22.9	25.7	24.3	9.7
CV	10.3	12.0	8.4	11.2
Trend	ns	Q *	Q *	ns
	Number of fruits per tree			
0	246	141	282	28
40	218	132	341	35
80	241	252	400	46
120	207	194	346	38
160	192	233	298	72
CV	18.7	33.6	18.9	42.3
Trend	ns	Q *	Q *	L *
	Average fruit weight (g)			
0	98.6	121.2	66.6	136.8
40	103.6	128.5	71.9	133.9
80	110.2	113.1	79.4	141.0
120	108.3	114.5	73.9	141.2
160	113.0	101.4	75.3	123.1
CV	10.3	12.0	8.4	11.2
Trend	ns	ns	Q *	ns

CV, ns, *, L, Q: coefficient of variation, non-significant, significant at 0.05 level, linear and quadratic trend of the response, respectively.

**Table 3:** Scores recorded for the association between previously normalized features and target variables by using the univariate regression method for ranking purposes.

Target Variable	Features													
	Ch *	GDD2	R **	N Dose	N	P	K	Ca	Mg	B	Cu	Zn	Mn	Fe
Yield	47.1	1.8	2.6	1.1	13.8	0.2	11.8	1.0	19.6	18.8	70.6	1.4	12.0	0.3
Skin firmness	11.5	23.4	10.8	1.3	0.1	40.3	0.4	14.9	1.0	1.5	1.7	18.5	0.1	2.7
Pulp firmness	164.1	72.7	0.3	0.4	0.3	70.5	3.3	10.7	8.6	0.4	10.1	20.4	<0.1	15.1
Fruit hue	107.6	37.7	38.8	<0.1	0.1	7.6	26.0	13.0	79.6	41.2	19.6	8.6	<0.1	7.7
Fruit chroma	12.9	<0.1	46.7	1.6	1.7	2.4	0.1	2.7	1.6	0.5	3.1	2.6	4.1	29.0
TSS	6.4	4.0	160.9	0.2	5.7	1.4	0.6	24.8	2.0	0.1	12.2	0.2	5.4	16.4
Total acidity	0.5	56.9	5.4	0.1	<0.1	54.8	2.7	9.0	22.7	29.4	9.7	28.7	0.1	0.9
Phenolics	5.7	0.3	1.3	0.2	3.5	0.7	4.3	4.3	2.3	2.4	8.5	<0.1	2.4	0.1
Carotenoids	85.4	2.0	0.5	5.1	3.0	15.0	1.5	2.7	5.6	2.6	13.4	3.9	2.4	24.5
Antioxidants	22.2	18.1	3.0	0.9	5.9	5.4	14.7	0.9	37.6	43.0	101.6	15.6	9.8	0.6

* Chill hours; ** Rainfall.

**Table 4:** KNN and SGD model results relating climatic and nutrient features to target variables in classification mode.

Target Variable	Cutoff Value	KNN	SGD	AUC	CA	TN	TP	FN	FP
Yield	16 ton ha^−1^	-	X	0.955	0.975	58	20	2	0
Skin firmness	8.82 N	-	X	0.892	0.925	56	18	4	2
Pulp firmness	2.5 N	-	X	0.949	0.950	39	18	1	2
Fruit hue	80	-	X	0.948	0.950	59	17	1	3
Fruit chroma	50	-	X	0.887	0.925	54	19	5	2
TSS	10.5 mg per 100 g	X	-	0.947	0.950	59	17	1	3
TTA	0.9%	X	-	0.728	0.738	49	10	13	8
Phenolics	280	-	X	0.625	0.662	10	37	11	22
Carotenoids	5.9	X	-	0.926	0.938	17	58	3	2
TAC	1800 mg per 100 g	-	X	0.955	0.975	58	20	2	0

TSS = total soluble solids; TTA = total titratable acidity; TAC = total antioxidant content; KNN = k-nearest neighbors; SGD = stochastic gradient decent; AUC = area under curve; CA = classification accuracy = (TN + TP)/total; TN = true negative specimens; TP = true positive specimens; FN = false negative specimens; FP = false positive specimens.

**Table 5:** Mean and standard deviation recorded for clr values of foliar nutrient components, for true negative specimens of target variables, by using the 2014–2017 data set. TSS = total soluble solids; TTA = total titratable acidity; TAC = total antioxidant content.

Feature	Yield	Skin Firmness	Pulp Firmness	Hue	Chroma	TSS	TTA	TAC
	clr mean ± standard deviation for true negative specimens							
N	3.16 ± 0.108	3.07 ± 0.425	3.13 ± 0.104	3.12 ± 0.118	3.12 ± 0.122	3.12 ± 0.118	3.15 ± 0.108	3.16 ± 0.109
P	0.866 ± 0.420	0.76 ± 0.386	0.90 ± 0.102	0.73 ± 0.279	0.81 ± 0.415	0.85 ± 0.420	1.06 ± 0.247	0.88 ± 0.412
K	3.06 ± 0.183	2.93 ± 0.400	2.91 ± 0.156	2.93 ± 0.142	3.01 ± 0.200	3.03 ± 0.210	3.04 ± 0.226	3.07 ± 0.184
Ca	2.70 ± 0.168	2.66 ± 0.380	2.69 ± 0.136	2.667 ± 0.134	2.74 ± 0.160	2.75 ± 0.156	2.75 ± 0.161	2.71 ± 0.165
Mg	1.27 ± 0.330	1.40 ± 0.336	1.60 ± 0.197	1.5 ± 0.174	1.37 ± 0.387	1.35 ± 0.410	1.28 ± 0.407	1.27 ± 0.335
Cu	−5.10 ± 0.209	−5.22 ± 0.846	−5.45 ± 0.428	−5.41 ± 0.358	−5.37 ± 0.421	−5.36 ± 0.416	−5.21 ± 0.407	−5.10 ± 0.211
Fe	−2.70 ± 0.228	−2.63 ± 0.438	−2.83 ± 0.197	−2.72 ± 0.223	−2.61 ± 0.171	−2.63 ± 0.173	−2.76 ± 0.208	−2.71 ± 0.230
Mn	−2.63 ± 0.169	−2.52 ± 0.408	−2.58 ± 0.165	−2.57 ± 0.161	−2.561 ± 0.179	−2.56 ± 0.174	−2.61 ± 0.175	−2.63 ± 0.167
Zn	−3.69 ± 0.308	−3.50 ± 0.571	−3.64 ± 0.158	−3.54 ± 0.193	−3.61 ± 0.301	−3.63 ± 0.299	−3.78 ± 0.242	−3.70 ± 0.302
B	−3.57 ± 0.181	−3.41 ± 0.531	−3.42 ± 0.137	−3.41 ± 0.126	−3.48 ± 0.219	−3.50 ± 0.226	−3.56 ± 0.224	−3.57 ± 0.180
F_v_	6.62 ± 0.087	6.49 ± 0.884	6.68 ± 0.072	6.65 ± 0.077	6.59 ± 0.061	6.59 ± 0.059	6.63 ± 0.087	6.62 ± 0.089
	clr back-transformed concentration ranges for true negative specimens							
	g kg^−1^							
N	28–30	27–28	25–29	25–30	25–32	25–32	27–29	28–30
P	1.2–6.8	2.4–4.9	2.7–3.1	1.5–4.2	1.1–7.0	1.2–7.4	2.3–5.3	1.3–6.8
K	21–33	18–35	17–27	19–26	18–36	18–38	18–36	21–33
Ca	15–22	17–22	15–20	15–20	15–25	16–25	16–23	15–22
Mg	2.3–8.2	2.0–10.9	4.2–8.0	4.5–7.4	2.1–11.4	1.9–11.9	1.9–10.0	2.3–8.1
	mg kg^−1^							
Cu	5–10	2–16	2–13	3–11	2–15	2–15	3–15	5–10
Fe	56–115	57–101	49–95	54–114	70–119	68–118	56–103	56–115
Mn	72–106	71–111	68–111	74–113	72–128	73–127	71–110	72–105
Zn	17–58	19–40	24–48	26–46	18–62	18–60	19–41	17–52
B	27–43	25–49	32–44	35–44	26–57	25–57	26–54	27–42

## Data Availability

The data presented in this study are available within the article.

## References

[1] Penso G.A., Santos C.E.M., Bruckner C.H., Costa J.C.F., Citadin I. (2018). Consumption, preferences and habits of purchasing consumers of peaches and nectarines. Rev. Bras. Frutic..

[2] Belisle C., Pha U.T.X., Adhikari K., Chavez D.J. (2018). A Fruit Quality Survey of Peach Cultivars Grown in the Southeastern United States. HortTechnology.

[3] Brown A.F., Yousef G.G., Guzman I., Chebrolu K.K. (2014). Variation of Carotenoids and Polyphenolics in Peach and Implications on Breeding for Modified Phytochemical Profiles. J. Amer. Soc Hort. Sci..

[4] Ricce W.S., Pandolfo C., Souza A.L.K., Massignam A.M., Vianna L.F.N. (2018). Análise de Riscos Climáticos Para as Culturas do Pessegueiro, Nectarineira e Ameixeira no Estado de Santa Catarina.

[5] Nienow A.A., Floss L.G. (2003). Peach and nectarine yields in the medium planes of Rio Grande do Sul, Brazil in years of soft winter. Cien. Rural..

[6] Castro L.A.S. (2010). Cultivares de Pessegueiro e Nectarineira com Alta Sanidade da Embrapa Clima Temperado.

[7] Schimtz J.D., Bianchi V.J., Pasa M., Da S., de Souza A.L.K., Fachinello J.C. (2012). Vigor and productivity of “Chimarrita” peach tree on different rootstocks. R. Bras. Agrociência.

[8] Mayer N.A., Ueno B., das Neves T.R., Rickes T.B. (2019). Five years of evaluation of rootstock effects on tree production, yield and yield efficiency of ‘Maciel’ peach tree. Rev. Fac. Agron..

[9] Byrne D.H. (2005). Trends in stone fruit cultivar development. HortTechnology.

[10] Mercier V., Bussi C., Plenet D., Lescourret F. (2007). Effects of limiting irrigation and of manual pruning on brown rot incidence in peach. Crop Prot..

[11] Montanaro G., Dichio B., Bati C.B., Xiloyannis C. (2012). Soil management affects carbon dynamics and yield in a Mediterranean peach orchard. Agric. Ecosyst. Environ..

[12] Dolinski M.A., Serrat B.M., Motta A.C.V., Cuquel F.L., Souza S.R., May-de Moi L.L., Monteiro L.B. (2005). Production, leaves concentration and fruit quqlity of the peach orchard “Chimarrita” as funciton of N fertilization at Lapa region, Parana state–Brazil. Rev. Bras. Frutic..

[13] Brunetto G., Melo G.W., Kaminski J., Ceretta C.A. (2007). Nitrogen fertilization in serial cycles and its impact on yield and quality of peach. Pesq. Agropec. Bras..

[14] Baldi E., Toselli M., Marangoni B. (2010). Nutrient partitioning in potted peach (*Prunus persica* L.) trees supplied with mineral and organic fertilizers. J. Plant Nutr..

[15] Baldi E., Toselli M. (2013). Root growth and survivorship in cow manure and compost amended soils. Plant Soil Environ..

[16] Melo G.W.B., Sete P.B., Ambrosini V.G., Freitas R.F., Basso A., Brunetto G. (2016). Nutritional status, yield and composition 3- of peach fruit subjected to the application of organic compost. Acta Sci..

[17] Famiani F., Bonghi C., Chen Z.H., Drincovich M.F., Farinelli D., Lara M.V., Proietti S., Rosati A., Vizzotto G., Walker R.P. (2020). Stone Fruits: Growth and Nitrogen and Organic Acid Metabolism in the Fruits and Seeds—A Review. Front. Plant Sci..

[18] Saenz J.L., DeJong T.M., Weinbaum S.A. (1997). Nitrogen stimulated increases in peach yields are associated with extended fruit development period and increased fruit sink capacity. J. Amer. Soc. Hortic. Sci..

[19] Gomez L., Vercambre G., Jordan M.O. (2020). Spatial-temporal management of nitrogen and carbon on the peach tree (*Prunus persicae* L. Batsch.). Sci. Hortic..

[20] Bravo K., Baldi E., Marcolini G., Sorrenti G., Cellini A., Quartieri M., Toselli M. (2015). Response of Hybrid Peach £ Almond Trees to Increasing Rate of Soil-Applied Urea and Compost Nitrogen. Compost. Sci. Util..

[21] Pascual M., VIllarb J.M., Rufat J. (2016). Water use efficiency in peach trees over a four-years experiment onthe effects of irrigation and nitrogen application. Agric. Water Manag..

[22] Ferreira L.V., Picolotto L., Gonçalves M.A., Valgas R.A., Antunes L.E.C. (2018). Fertilizer maintenance nitrogen in vegetative development and production of peach. Braz. J. Agric..

[23] Rubio Ames Z., Brecht J.K., Olmstead M.A. (2020). Nitrogen fertilization rates in a subtropical peach orchard: Effects on tree vigor and fruit quality. J. Sci. Food Agric..

[24] Barreto C.F., Ferreira L.V., Navroski R., Frasson S.F., Cantillano R.F.F., Vizzotto M. (2017). Adubação nitrogenada em pessegueiros (*Prunus persica* (L.) Batsch): Influência sobre a qualidade pós-colheita. Rev. Iber. Tecnol. Postcosecha.

[25] Dolinski M.A., Dangelo J.W., Cuquel F.L., Motta A.C.V., Mio L.L.M. (2018). Quality peach produced in fertilizer doses of nitrogen and green pruning. Bragantia.

[26] Ferreira L.V., Picolotto L., Pereira I.S., Schmitz J.D., Antunes L.E.C. (2018). Nitrogen fertilization in consecutive cycles and its impact on high-density peach crops. Pesq. Agropec. Bras..

[27] Toselli M., Baldi E., Cavani L., Mazzon M., Quartieri M., Sorrenti G., Marzadori C. (2019). Soil-plant nitrogen pools in nectarine orchard in response to long-term compost application. Sci. Total Environ..

[28] Mia M.J., Monaci E., Murri G., Massetani F., FacchI J., Neri D. (2020). Soil Nitrogen and Weed Biodiversity: An Assessment under Two Orchard Floor Management Practices in a Nitrogen Vulnerable Zone in Italy. Horticulturae.

[29] Cui M., Zeng L., Qin W., Feng J. (2020). Measures for reducing nitrate leaching in orchards: A review. Environ. Pollut..

[30] Gomes F.R.C., Fachinello J.C., Medeiros A.R.M., Giacobbo C.L., Santos I.P. (2005). Influence of the soil management and fruit thinning intensity on the growth and quality of peaches ‘Cerrito’ and ‘Chimarrita’. Rev. Bras. Frutic..

[31] Souza F., Alves E., Pio R., Castro E., Reighard G., Freire A.I., Mayer N.A., Pimentel R. (2019). Influence of Temperature on the Development of Peach Fruit in a Subtropical Climate Region. Agronomy.

[32] Brunetto G., Ceretta C.A., Kaminski J., Melo G.M., Girotto E., Trentin E.E., Lourenzi C.R., Vieira R.C., Gatiboni L.C. (2009). Produção e composição química da uva de videiras Cabernet Sauvignon submetidas à adubação nitrogenada. Ciênc. Rural..

[33] Frene J.P., Frazier M., Rutto E., Jones M., Clark B., Parker M., Terrence G.G. (2020). Early response of organic matter dynamics to pinebiochar in sandy soil under peach trees. Agrosyst. Geosci. Environ..

[34] Lorensini F., Ceretta C.A., Conti L.D., Ferreira P.A.A., Dantas M.K.L., Brunetto G. (2017). Nitrogen fertilization in the growth phase of Chardonnay and Pinot Noir vines and nitrogen forms in sandy soil of the Pampa Biome. Rev. Ceres..

[35] Barreto C.F., Antunes L.E.C., Ferreira L.V., Navroski R., Benati J.A., Nava G. (2020). Nitrogen fertilization and genotypes of peaches in high-density. Rev. Bras. Frutic..

[36] Righetti T., Wilder K.L., Cummings G. (1990). Plant analysis as an aid in fertilizing orchards. Soil Testing and Plant Analysis.

[37] CQFS—Comissão de Química e Fertilidade do Solo (2016). Manual de Calagem e Adubação Para os Estados de Rio Grande do Sul e de Santa Catarina.

[38] Parent S.É. (2020). Introduction to Machine Learning for Ecological Engineers, Nextjournal. https://nextjournal.com/essicolo/cc2020?change-id=Cmh2rwpPcYJ6zXzuu2czyh.

[39] Betemps D.L., Paula B.V., Parent S.É., Galarça S.P., Mayer N.A., Marodin G.A.B., Rozane D.E., Natale W., Melo G.W.B., Parent L.E. (2020). Humboldtian diagnosis of peach tree (*Prunus persica*) nutrition using machine-learning and compositional methods. Agronomy.

[40] Parent L.E., Jamaly R., Atucha A., Parent E.J., Workmaster B.A., Ziadi N., Parent S.É. (2021). Current and next-year cranberry yields predicted from local features and carryover effects. PLoS ONE.

[41] Soil Survey Staff (2014). Keys to Soil Taxonomy.

[42] Alvares C.A., Stape J.L., Sentelhas P.C., Gonçalves J.L.M., Sparovek G. (2013). Koppen’s climate classification map for Brazil. Meteorol. Z.

[43] AGROMET Meteorological Data from Pelotas-RS in Real Time. http://agromet.cpact.embrapa.br/online/Resumos_Mensais.htm.

[44] Anderson N.A. (2011). Diversity of Low Chill Peaches (*Prunus Persica*) from Asia, Brazil, Europe and the USA. Ph.D. Thesis.

[45] Comissão de Química e Fertilidade do Solo (CQFS RS/SC) (2004). Manual de Adubação e de Calagem Para os Estados do Rio Grande do Sul e Santa Catarina.

[46] Hoying S.A., Robinson T.L., Anderson R.L. (2007). More productive and profitable peach planting systems. N. Y. Fruit Q..

[47] Tedesco M.J., Gianello C., Bissani C.A., Bohnen H., Volkweiss S.J. (1995). Analysis of Soils, Plants and Other Materials.

[48] Silva F.C. (2009). Manual de Análises Químicas de Solos, Plantas e Fertilizantes.

[49] Barreto C.F., Navroski R., Cantillano R.F.F., Vizzotto M., Nava G. (2019). Potassium fertilization on quality of peaches. Rev. Ciênc. Agrá..

[50] Swain T., Hills W.E. (1959). The phenolic constituents of Punnus domestica. I.—The quantitative analysis of phenolic constituents. J. Sci. Food Agric..

[51] Mitcham B., Cantwell M., Kader A. (1996). Methods for Determining Quality of Fresh Commodities.

[52] Talcott T.S., Howard R.L. (1999). Phenolic autoxidation is responsible for color degradation in processed carrot puree. J. Agric. Food Chem..

[53] Brand-Williams W., Cuvelier M.E., Berset C. (1995). Use of a free radical method to evaluate antioxidant activity. LWT-Food Sci. Technol..

[54] Morandi B., Manfrini L., Losciale P., Zibordi M., Corelli-Grappadelli L. (2010). The positive effect of skin transpiration in peach fruit growth. J. Plant Physiol..

[55] Grossman Y.L., DeJong T.M. (1995). Maximum fruit growth potential and seasonal patterns of resource dynamics during peach growth. Ann. Bot..

[56] McMaster G.S., Wilhelm W.W. (1997). Growing degree-days: One equation, two interpretations. Agric. Meteorol..

[57] SAS Institute (1996). The SAS-System for Windows: Release 6.08.

[58] Plummer M., Stukalov A., Denwood M. (2016). Rjags: Bayesian Graphical Models Using MCMC (Version 4–6).

[59] R Core Team A Language and Environment for Statistical Computing; R Foundation for Statistical Computing. https://www.R-project.org/.

[60] Gelman A., Hill J. (2007). Data Analysis Using Regression and Multilevel/Hierarchical Models.

[61] Milatovic D., Nikolic D., Durovic D. (2010). Variability, heritability and correlatins of some factors affctig productiity in peach. Hortic. Sci..

[62] Galarça S.P., Fachinello J.C., Betemps D.L., Hoffmann A., Marodin G.A.B., Pretto A., Nunes F.S., Dias F.P. (2013). Crescimento e desenvolvimento de pessegueiros ‘Chimarrita’ e ‘Maciel’ sobre diferentes porta-enxertos e locais de cultivo. Cienc. Rural..

[63] Raseira M.C.B. (2016). Breeding for low-chill ares. Workshop on Temperate Fruit Trees Adaptation in Subtropical Areas.

[64] Raseira M.C.B., Franzon R.C., Feldberg N.P., Scaranari C., Pereira J.F.M. (2020). BRS Jaspe: A processing peach cultivar for low chill areas. Crop Breed. Appl. Biotechnol..

[65] Melo G.W., Rozane D.E., Brunetto G., Lattuada D.S. (2018). Doscrimiinant analysis in the selection ofgroups of peach cultivars. Acta Hortic..

[66] Rufat J., DeJong T.M. (2001). Estimating seasonal nitrogen dynamics in peach trees in response to nitrogen availability. Tree Physiol..

[67] Parent S.-É., Lafond J., Paré M.C., Parent L.E., Ziadi N. (2020). Conditioning Machine Learning Models to Adjust Lowbush Blueberry Crop Management to the Local Agroecosystem. Plants.

[68] Rutowski K., Michalczuk B., Konopacki P. (2008). Nondestructive determination of ‘Golden Delicious’ apple quality and harvest maturity. J. Fruit Ornament. Plant Res..

[69] Rooban R., Shanmugam M., Venkatesan T., Tamilmani C. (2016). Physiochemical changes during different stages of fruit ripening of climacteric fruit of mango (*Mangifera indica* L.) and non-climacteric of fruit cashew apple (*Anacardium occidentale* L.). J. Appl. Adv. Res..

[70] Mira L., Fernandez M.T., Santos M., Rocha R., Florencio M.H., Jennings K.R. (2002). Interactions of flavonoids with iron and copper ions: A mechanism for their antioxidant activity. Free Rad. Res..

[71] Symonowicz M., Kolanek M. (2012). Flavonoids and their properties to form chelate complexes. Biotechnol. Food Sci..

[72] May-De-Mio L.L., TutidA I., Motta A.C., Dolinski M.A., Serrat B.M., Monteguti D. (2008). Doses de aplicação de nitrogênio e potássio em relação à podridão parda e sarna em ameixeira ‘Reubennel’ na região de Araucária, Paraná. Trop. Plant Pathol..

[73] Fernandez-Escobar R., OrtiZ-Urquiza A., Prado M., Rapoport H.F. (2008). Nitrogen status influence on olive tree flower quality and ovule longevity. Environ. Exp. Bot..

[74] IBGE-Instituto Brasileiro de Geografia e Estatística (2019). Produção Agrícola Municipal.

[75] Nava G., Dechen A.R. (2009). Long-term annual fertilization with nitrogen and potassium affect yield and mineral composition of ‘Fuji’ apple. Sci. Agric..

[76] Bussi C., Genard M., Horticoles S., Avignon F. (2014). Thinning and pruning to overcome alternate bearing in peach trees. Eur. J. Hortic. Sci..

[77] Taylor B.K., van den Ende B. (1969). The Nitrogen Nutrition of the Peach Tree. IV. Storage and Mobilization of Nitrogen in Mature Trees. Aust. J. Agric. Res..

[78] Tromp J. (1983). Nutrient reserves in roots of fruit trees, in particular carbohydrates and nitrogen. Plant Soil..

[79] Tagliavini M., Millard P., Quartieri M., Marangoni B. (1999). Timing of nitrogen uptake affects winter storage and spring remobilisation of nitrogen in nectarine (*Prunus persica* var. nectarina) trees. Plant Soil..

[80] Tagliavini M., Zavalloni C., Rombolà A.D., Quartieri M., Malaguti D., Mazzanti F., Millard P., Marangoni B. (2000). Mineral Nutrient Partitioning to Fruits of Deciduous Trees. Acta Hortic..

[81] Cruz A.F., de Almeida G.M., Wadt P.G.S., de Carvalho P.M., Ramo M.L.G. (2019). Seasonal Variation of Plant Mineral Nutrition in Fruit Trees. Braz. Arch. Biol. Technol..

[82] Rumpel C., Creme A., Ngo P.T., Velasquez G., Mora M.L., Chabbi A. (2015). The impact of grassland management on biogeochemical cycles involving carbon, nitrogen and phosphorus. J. Soil Sci. Plant Nutr..

[83] Brunetto G., Lorensini F., Ceretta C.A., Ferreira P.A.A., Couto R.R., De Conti L., Ciotta M.N., Kulmann M., Schneider O., Somavilla L.M. (2017). Contribution of mineral N to young grapevine in the presence or absence of cover crops. J. Soil Sci. Plant Nutr..

[84] Kader A.A. (1995). Fruit Maturity, Ripening, and Quality Relationships. International Symposium Effect of Pre- & Postharvest Factors in Fruit Storage 485.

[85] Cirilli M., Bassi D., Ciacciulli A. (2016). Sugars in peach fruit: A breeding perspective. Hortic. Res..

[86] Testoni A. Momento di racolta, qualita, condizionamento e confezionamento delle pesche. Proceedings of the Symposium la Peschicoltura Veronesa Alle Soglie del.

[87] Ventura M., Sama A., Minguzzi S., Lanzon S., Sansavini S. (2000). Ottimizzazione del carico di fruit per migliorare la produzione e la qualita delle nectarine Supercrimson e Venus. Proc. XXIV Conv. Peschiolo.

[88] Hilaire C. The peach industry in France: State of art, research and development. Proceedings of the First Mediterranean Peach Symposium.

[89] Crisosto C.H., Mitchell F.G., Ju Z. (1999). Susceptibility to chilling injury of peach, nectarine and plum cultivars grown in California. Hort. Sci..

[90] Culpepper C.W., Caldwell J.S. (1930). The Canning Quality of Certain Commercially Important Eastern Peaches.

